# Clustering time trends of breast cancer incidence in Africa: a 27-year longitudinal study in 53 countries

**DOI:** 10.4314/ahs.v21i1.8

**Published:** 2021-03

**Authors:** Mehran Saberian, Kamran Mehrabani, Hadi Raeisi Shahraki

**Affiliations:** 1 Student Research Committee, Shahrekord University of Medical Sciences, Shahrekord, Iran Address: Rahmatieh educational complex, Shahrekord University of Medical Sciences, Shahrekord, Iran. Mehran_saberian@yahoo.com; 2 Department of Biostatistics, Faculty of medicine, Shiraz University of medical sciences, Shiraz, Iran Address: Shiraz University of Medical Sciences, Zand Avenue, Shiraz, Iran. kamran.mehrabani@gmail.com; 3 Department of Epidemiology and Biostatistics, Faculty of Health, Shahrekord University of Medical Sciences, Shahrekord, Iran

**Keywords:** Africa, breast cancer, incidence, latent mixture model, trend

## Abstract

**Background:**

Breast cancer is the most common, frequently diagnosed cancer with the highest incidence among female worldwide. Although the incidence is decreasing in developed countries, it is on increase in most of the African countries.

**Objective:**

This study aimed to identify different time trends of breast cancer incidence among African countries using latent mixture approach.

**Methods:**

The information includes newly diagnosed breast cancer patients per 100,000 women for 53 African countries in a period of 1990–2016. Latent mixture modeling was performed in Mplus 7.4 software.

**Results:**

The overall trend of breast cancer in Africa was increasing. Latent mixture model with 5 clusters was estimated as the best using fit indices and linear growth trajectories were specified for each cluster. Nigeria was the only country which belongs to a cluster with negative slope indicating a slow decrease in the breast cancer incidence; also, Seychelles was the only country that showed a sharp increase over time. 31 countries belonged to a cluster with a slope of 0.08, indicating that the incidence of breast cancer is almost constant over time. Cluster 3 including Algeria, Angola, Botswana, Central African Republic, Cote d'lvoire, Equatorial Guinea, Lesotho, Libya, Namibia, Somalia, Sudan, Swaziland, Uganda and Zimbabwe and cluster 2 including Gabon, Mauritius, Morocco, South Africa, Tunisia and Congo showed a slow and moderate increase in the incidence of breast cancer, respectively.

**Conclusion:**

Providing health education programs is essential in African countries with rising trend of breast cancer during the last decades.

## Introduction

Annually 1.3 million cancer patients are identified worldwide. At present, cancer is known as one of the most important issues in public health because in developed countries more than 50% of the burden of diseases is related to cancer and in developing countries the incidence of cancer is increasing 1. Breast cancer is the most common and frequently diagnosed cancer with the highest incidence and eminent cause of cancer death among females worldwide 2–7.

In year 2012, 1.7 million cases and more than 500,000 deaths due to breast cancer have been reported^5^, ^6^,^8^. Although the incidence is decreasing in developed countries, breast cancer is increasing in most of the African countries^8^, ^9^. The International agency for cancer research (IARC) announced that the incidence rate of female's breast cancer is 39 per 100 000 in southern Africa and 27 per 100 000 in central Afria in 2012 2. The burden of breast cancer will be double in Africa by 2030 because of the increase in the population growth and unhealthy lifestyle 2.

Recently, modeling time trends in health sciences has been more attentive. Analyzing time trends requires extensive datasets including repeated measures in a longitudinal design and also a practical model to describe complex growth patterns^10^. Latent mixture approach as the most appropriate model in this setting tries to capture unobserved heterogeneity, using categorical latent variables^10^, ^11^. In the current manuscript, we call this categorical latent variable as cluster. Despite the numerous studies about the incidence and mortality of breast cancer in African countries, lack of studies about heterogeneity of breast cancer temporal trends among African countries is obvious. Therefore, this study aimed to identify different time trends of breast cancer incidence among African countries using latent mixture model approach.

## Materials and methods

The information including newly diagnosed breast cancer patients per 100,000 women for 53 African countries which was reported in Gapminder web site (Stockholm, Sweden. Freely available at www.gapminder.org/data) in a period of 1990–2016 was collected.

Estimation of number of clusters is the most important issue in latent mixture modeling. To address this problem, we used likelihood ratio test and due to small sample size, P-value <0.1 was considered as statistically significant. Latent mixture modeling was performed in Mplus 7.4 software which uses maximum likelihood estimator based on EM algorithm.

## Results

**Figure 1:**
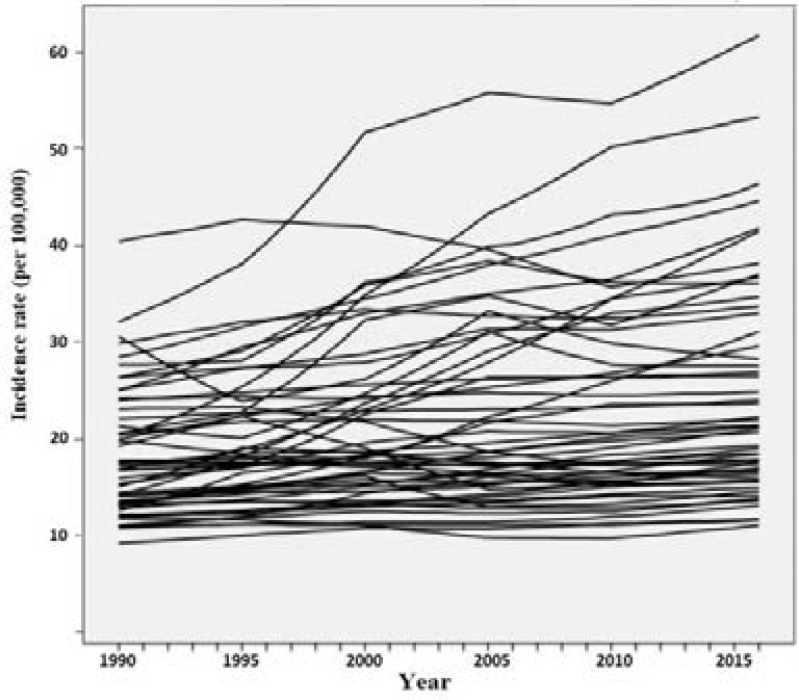
Breast cancer incidence rate trajectories of 53 African countries

Descriptive statistics of breast cancer incidence rate for African countries from 1990 to 2016 (in 5-year intervals) are summarized in Table 1 and growth trajectory for each country is displayed in Figure 1. Although these rates indicate an overall increasing trend, trajectories show different trends among African countries so latent mixture modeling was utilized to divide the countries into different clusters based on their incidence trend over time.

**Table 1:** Descriptive statistics of breast cancer incidence rates (per 100000) for 53 African countries

Year	Min	Max	Mean	SD	Median
1990	9.2	40.4	18.3	6.7	16.8
1995	10.0	42.7	19.6	7.1	17.6
2000	10.8	51.7	21.4	8.7	18.4
2005	9.8	55.8	22.6	10.0	18.7
2010	9.7	54.7	23.1	10.3	19.8
2016	11.1	61.7	24.6	11.4	21.1

**Figure 2:**
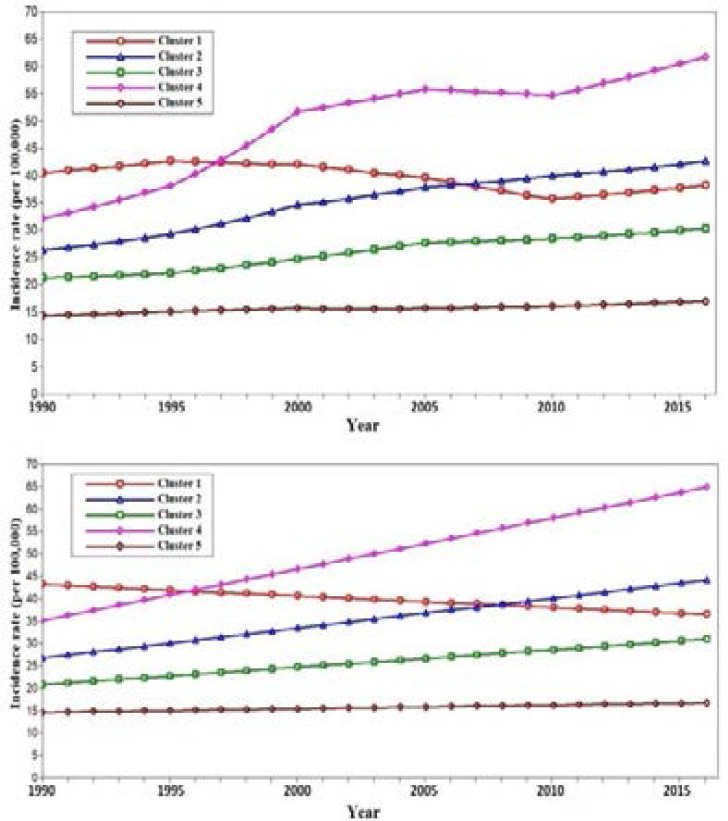
The overall mean (top) and estimated linear trend (bottom) of each cluster

Goodness of fit indices for different clusters of latent mixture model were summarized in Table 2 and 5-cluster model was selected as the best based on the solutions of likelihood ratio test (P-value=0.08). Based on 5-cluster model, linear growth trajectories with different intercepts and trends were specified for all of the clusters, as reported in Table 3. Besides, the entropy of 0.89 revealed good quality for the membership of latent classes. Overall mean and estimated linear trend of each trajectory are displayed in Figure 2.

**Table 2:** The goodness of fit indices for different clusters of latent mixture model

Fit indices	Number of clusters						
	1	2	3	4	5	6	7
AIC	10347	8826	8142	7809	7673	7482	7363
BIC	10404	8889	8211	7884	7754	7569	7456
SSBIC	10313	8789	8101	7765	7625	7430	7308
LRT P-value	---	0.48	0.49	0.28	0.08	0.23	0.23

AIC: Akaike information criterion, BIC: Bayesian information criterion, SSBIC: sample size adjusted Bayesian information criterion, LRT: likelihood ratio test

**Table 3:** Results of growth mixture models for clustering of African countries based on breast cancer incidence rates

Cluster	Number of countries	Intercept		Slope	
		Estimate	SE	Estimate	SE
1	1	43.18	0.13	-0.26	0.01
2	6	26.73	1.45	0.67	0.18
3	14	20.78	1.11	0.39	0.08
4	1	35.12	0.44	1.15	0.03
5	31	14.60	0.58	0.08	0.03

Nigeria was the only country which belonged to cluster 1 with negative slope indicating slow decrease in breast cancer incidence rate over time. Cluster 2 (including Gabon, Mauritius, Morocco, South Africa, Tunisia and Congo) and cluster 3 (including: Algeria, Angola, Botswana, Central African republic, Cote d'lvoire, Equatorial Guinea, Lesotho, Libya, Namibia, Somalia, Sudan, Swaziland, Uganda and Zimbabwe) and can be defined as having a moderate and slow increase in the incidence rate over time, respectively.

Seychelles was the only country which belonged to cluster 4 with slope 1.15 indicating a sharp increase over time and other 31 countries belonged to cluster 5 with a slope of 0.08, indicating that incidence of breast cancer is almost constant over time (Table 3).

## Discussion

Breast cancer is a common cancer among African nations. Reported results clustered 53 African countries into 5 clusters with different incidence pattern of breast cancer. Nigeria was the only country with decreasing trend of incidence and Seychelles experienced very sharp increasing trend. The incidence was increased moderately in Gabon, Mauritius, Morocco, South Africa, Tunisia and Congo. Moreover, 14 countries including Algeria, Angola, Botswana, Central African Republic, Cote d'lvoire, Equatorial Guinea, Lesotho, Libya, Namibia, Somalia, Sudan, Swaziland, Uganda and Zimbabwe had a slow increase during last decades, and the incidence rate among another 31 African countries was almost constant.

Decreasing trend of BC in Nigeria was confirmed at a few previous studies. For example, in a study done in 4 hospitals in Eastern Nigeria, the decreasing trend of BC was reported during 1960 to 2008 12. Some studies indicated that there was no specific path for incidence of BC in Nigeria. We can see an increase during 2008 to 2009 and a decrease in 2010. In 2010 to 2013, the incidence was increased and again from 2013 to 2015 it was decreased^13^. The difference between the aforementioned study and our findings might be due to assessment of an overall long term trend in the current study. There were also some related articles which have reported different patterns in different population or periods of time. Elima et al. analyzed data from 2 population Ibadan and Abuja in two years (2009 to 2010) and showed that the incidence of BC as increased but cervical cancer had stable incidence 14. Besides, the results of a research on black African population in Nigeria showed that the incidence rate was 63.7 to 130.9 in two periods (1971–1980, 1981–1990). The incidence rose in 1978 because of the low level of the people's awareness and negligence of the consultant. Also, in 1982 in collaboration with TV and radio for increasing awareness, we see raising incidence and in 1986 because of development of economic conditions BC incidence decreased. In 1987, because of the use of radiotherapy in the hospital Ibadan, the incidence rate increased^15^. Although studies about the incidence of BC in Seychelles is rare, the observed sharp increasing trend may be due to greater proportion of early stage diagnosis as a results of development and better screening compared to other African countries 16.

Our findings about moderate increase of incidence in Gabon, Mauritius, Morocco, South Africa, Tunisia and Congo are consistent with most of the related articles in these countries. Khalis et al. showed that BC incidence increased from 35 to 39 per 100000 women during 2004 to 2008 in Morocco 5. Another study which was done at the eastern morocco announced BC incidence was increased from 23 to 36.8 between 2006–2012 because of some reasons like late age of marriage, reducing fertility, change in diet, physical activity and change in prevalence of protective factors like age at the first child delivery and duration of breast feeding^17^, ^18^. The incidence of BC was increased in Tunisia from 23.4 to 28.3 per 100000 that showed annual increase of 2.2% from 1993 to 2006. This trend is due to change in lifestyle that increases the risk factors of BC, and shortage of knowledge about an effective screening program for breast cancer among Tunisian women^19^. Another study that showed the burden of BC in Tunisia revealed that BC incidence is increased with an annual rate of 2.5% as in 1993 it was almost 18 and in 2007 reached 40 per 100000 women^20^. Also, the incidence of BC in South Africa was reported by Singh et al. which increased from 25.1 to 26.9 per 100000 during 1994 to 2009. The observed patterns showed a rising trend among Black, Colored and Asian groups but a falling trend for white population^21^.

Formal statistics about the incidence of BC in Algeria from cancer registry of Setif confirmed the increasing trend from 10.4 in 1987–1989 to 24.8 in 2005–2007 which was slightly higher than the reported rate for cluster of Algeria in this study^22^. The observed increase may be due to the prevalence of risk factors like obesity, level of sex hormones, late child bring, having fewer child and unhealthy diet^23^. Slow increase of BC incidence was also demonstrated in Uganda; BC increased from 11.7 to 22.0 per 100000 during 1960 to 1997 24. This result may be associated with increase in overweight or obesity among women^25^, ^26^. The same study investigated the trend in the Zimbabwe. This study indicated that the breast cancer incidence in 1991–1995, 1996–2000 and 2001–2005 years was 20.9, 26.9 and 30.3, respectively 27. Besides, slow increase of BC aCentral Africa was suggested by Balekouzou et al. as the incidence rose from 11.0 to 14.8 per 100000 during 2003 to 2015 and the trend of incidence and mortality increased with age less than 45 years old^28^.

In line with our findings, Boder et al. reported a slow increase in the incidence of BC in Libya because of improving diagnostic practice (mammography, immunostaining) in the period of 2002 to 2006 29. However, in spite of the reported results about Sudan, a previous study in Khartoum (capital of Sudan) showed higher growth of BC incidence. The rate was 31.1 in 1966 and 34.1 per 100000 in 2000. It may be due to many risk factors like urbanization, early menarche, late childbearing, having fewer children, obesity or increasing awareness and detection 30. The study of Dey et al. in Egypt showed increasing and decreasing trends for urban and rural areas, respectively. Furthermore, we found that the incidence rate decreased in urban society, but in rural areas statistics showed an imperceptible increase in this period; this result is due to the reproductive risk factor^31^. The study in Yaounde in Cameroon revealed that the frequency of female breast cancer in 2004, 2006 and 2011 was 177, 175 and 163, respectively 32.

Using an advanced statistical model and investigation of longitudinal trajectories in a large scale are two key features of the current study. As a limitation, there was no related information about a few number of African countries. Investigation of the incidence of breast cancer in different age categories or mortality patterns of breast cancer in Africa is highly suggested for future studies.

## Conclusion

The incidence of breast cancer was increased in most of the African countries. Therefore, providing health education programs is essential in countries with rising trend of breast cancer during the last decades.
